# Diagnosis delay and follow-up strategies in colorectal cancer. Prognosis implications: a study protocol

**DOI:** 10.1186/1471-2407-10-528

**Published:** 2010-10-05

**Authors:** Salvador Pita Fernández, Sonia Pértega Díaz, Beatriz López Calviño, Paloma González Santamaría, Teresa Seoane Pillado, Francisco Arnal Monreal, Francesc Maciá, María Antonia Sánchez Calavera, Alejandro Espí Macías, Manuel Valladares Ayerbes, Alejandro Pazos, Margarita Reboredo López, Luis González Saez, María Ramos Montserrat, Josep María Segura Noguera, Isabel Monreal Aliaga, Luis González Luján, María Martín Rabadán, Cristiane Murta Nascimento, Olga Pueyo, Marta Maia Boscá Watts, Elena Cabeza Irigoyen, Montserrat Casmitjana Abella, Marina Pinilla, Ana Costa Alcaraz, Amador Ruiz Torrejón, Andrea Burón Pust, Concepción García Aranda, María de Lluc Bennasar, Sergio Lafita Mainz, Maite Novella, Hermini Manzano, Catalina Vadell, Esther Falcó, Magdalena Esteva

**Affiliations:** 1 Clinical Epidemiology and Biostatistics Unit, A Coruña Hospital, Hotel de Pacientes 7ª Planta, As Xubias 84, A Coruña, 15006, Spain; 2 Department of Anatomical Pathology, A Coruña Hospital, As Xubias 84, A Coruña, 15006, Spain; 3 Evaluation and Clinical Epidemiology Department, Hospital del Mar, Passeig Maritim 25-29, Barcelona, 08003, Spain; 4 Canal Imperial Primary Care Center, Paseo Colón 4, Zaragoza, 50006, Spain; 5 Department of Gastroenterology, University Clinic Hospital of Valencia, Avenida Blasco Ibáñez 17, Valencia, 46010, Spain; 6 Department of Oncology, A Coruña Hospital, As Xubias 84, A Coruña, 15006, Spain; 7 Department of Information and Communication Technologies, Computer Science Faculty, University of A Coruña, Campus de Elviña s/n, A Coruña, 15071, Spain; 8 Department of Surgery, A Coruña Hospital, As Xubias 84, A Coruña, 15006, Spain; 9 Deparment of Public Health, Balearic Department of Health, Font i Monteros s/n, Palma de Mallorca, 07003, Spain; 10 Lluis Saye Primary Care Centre, Torres I Amat 8, Barcelona, 08001, Spain; 11 Monreal del Campo Primary Care Centre, Pirineos 2, Monreal Del Campo, Teruel, 44300, Spain; 12 Serrería II Primary Care Centre, Valencia Institute of Health, Pedro de Valencia 26, Valencia, 46022, Spain; 13 Can Misses Primary Care Centre, Avinguda De La Pau s/n, Eivissa, 07800, Spain; 14 Aliaga Primary Care Centre, Polígono Industrial El Quiñón s/n, Teruel, 44150, Spain; 15 Health Plan Agency, Balearic Department of Health, Cecilio Metelo 18, Palma de Mallorca, 07003, Spain; 16 Clinical Epidemiology Unit, Hospital del Mar, Passeig Maritim 25-29, Barcelona, 08003, Spain; 17 Delicias Norte Primary Care Centre, Santa Orosia 46, Zaragoza, 50010, Spain; 18 Nazaret Primary Care Centre, Parque Nazaret 7, Valencia, 46024, Spain; 19 Functional Informatization Unit, Balearic Department of Health, Reina Esclaramunda 9, Palma de Mallorca, 07003, Spain; 20 Balearic Health System, Primary Care Management, Calle Mestre Perosi 17, Palma de Mallorca, 07008, Spain; 21 Pyrinees High Resolution Hospital, Calzada de Rapit s/n, Jaca, 22700, Spain; 22 Department of Gastroenterology, Hospital Can Misses, Carre Corona 32, Eivissa, 07800, Spain; 23 Department of Oncology, Hospital Son Dureta, Carre Andrea Dòria 55, Palma de Mallorca, 07014, Spain; 24 Department of Oncology, Hospital Manacor, Carretera Manacor-port d'Alcúdia s/n, Manacor, Mallorca, 07500, Spain; 25 Department of Oncology, Hospital Son Llatzer, Carretera Manacor km 4, Palma Mallorca, 07198, Spain; 26 Primary Health Care Research Unit, Primary Health Care Mallorca District, Balearic Health Service, Reina Esclaramunda 9, Palma de Mallorca, 07003, Spain

## Abstract

**Background:**

Controversy exists with regard to the impact that the different components of diagnosis delay may have on the degree of invasion and prognosis in patients with colorectal cancer. The follow-up strategies after treatment also vary considerably. The aims of this study are: a) to determine if the symptoms-to-diagnosis interval and the treatment delay modify the survival of patients with colorectal cancer, and b) to determine if different follow-up strategies are associated with a higher survival rate.

**Methods/Design:**

Multi-centre study with prospective follow-up in five regions in Spain (Galicia, Balearic Islands, Catalonia, Aragón and Valencia) during the period 2010-2012. Incident cases are included with anatomopathological confirmation of colorectal cancer (International Classification of Diseases 9th revision codes 153-154) that formed a part of a previous study (n = 953).

At the time of diagnosis, each patient was given a structured interview. Their clinical records will be reviewed during the follow-up period in order to obtain information on the explorations and tests carried out after treatment, and the progress of these patients.

Symptoms-to-diagnosis interval is defined as the time calculated from the diagnosis of cancer and the first symptoms attributed to cancer. Treatment delay is defined as the time elapsed between diagnosis and treatment. In non-metastatic patients treated with curative intention, information will be obtained during the follow-up period on consultations performed in the digestive, surgery and oncology departments, as well as the endoscopies, tumour markers and imaging procedures carried out.

Local recurrence, development of metastases in the follow-up, appearance of a new tumour and mortality will be included as outcome variables.

Actuarial survival analysis with Kaplan-Meier curves, Cox regression and competitive risk survival analysis will be performed.

**Discussion:**

This study will make it possible to verify if the different components of delay have an impact on survival rate in colon cancer and rectal cancer. In consequence, this multi-centre study will be able to detect the variability present in the follow-up of patients with colorectal cancer, and if this variability modifies the prognosis. Ideally, this study could determine which follow-up strategies are associated with a better prognosis in colorectal cancer.

## Background

In Spain, colorectal cancer is responsible for 12.6% of all deaths as a result of cancer in men, and 15.1% in women, according to data from 2007, representing the second most important tumour location in men and women [1]. It is estimated that in our country around 22,000 new cases are detected each year, with 13,500 deaths [2]. Survival of colorectal cancer at 5 years, according to data from EUROCARE 4 for cases diagnosed between 2000 and 2002, is 61.5% [3]. The survival rate is slightly higher amongst women than men, and in the location in the colon compared to the rectum [4]. In recent years, a 2% increase in the global survival rate has been recorded in Spain [5].

Colorectal cancer is usually diagnosed from clinical manifestations, as a result of a screening programme, or as a chance finding. Between the start of the illness and its diagnosis or treatment, there is a variable period of time known as diagnosis delay. Diagnosis delay may be affected by the characteristics of the illness, the characteristics of the patient, and the characteristics of the healthcare system. Studies carried out in our country reveal that the period of time between the appearance of the first symptoms and the first consultation in colorectal cancer is a median of 49 days [6].

Different factors associated with the patient or the healthcare system have been identified as modifiers of diagnosis delay [7-12]. Not recognising the severity of the symptoms, low socio-economic level, the location of the tumour, diagnostic errors and the application of inadequate tests or ones with previously negative results may all increase the delay. The factors associated with a shorter diagnosis delay include: associated comorbidity, visiting the hospital directly, or the use of derivation protocols. There is no clear evidence with regard to factors such as the patient's age or sex, the fear of being diagnosed with cancer, the existence of pain, educational level or family history. Neither is there any evidence that the frequency of use of the healthcare system by the patient or the use of fast access to endoscopies reduces diagnosis delay.

It appears reasonable to consider that reducing the delay could allow for diagnosis in earlier stages of the illness, and therefore improve the prognosis. However, the results are contradictory [13-20], as it has even been indicated that greater diagnosis delay is associated with a better prognosis [19] or that the delay is not associated with the stage or prognosis [18]. Two recent reviews made by members of this team reveal that there is controversy with regard to the role that delay plays in relation to the survival rate and degree of invasion in the diagnosis of colorectal cancer [21,22].

Once the tumour is diagnosed, the follow-up protocol for patients with cancer of the colon and rectum may modify the prognosis. The ideal recommendations in the follow-up process have yet to be described [23]. Different meta-analyses have been carried out on the effects of the follow-up on the prognosis of cancer of the colon and rectum [24-29]. The results of these meta-analyses coincide in that global survival is higher amongst patients who followed an intensive programme with respect to those who did not, and no improvement was seen in the specific survival rate. The survival rate was higher for those who used detection techniques such as tomography and carcinoembryonic antigen (CEA) determinations [25,26]. Intensive follow-up was associated with earlier detection of recurrences, the detection of recurrences susceptible to surgical resection and the probability of curative resection [26,27].

These follow-up programmes include frequent medical check-ups, CEA determinations, radiological studies of the thorax, abdominal ecography or tomography, and colonoscopy. However, the ideal frequency for carrying out these tests or the type of tests to be applied is not clear. The hypothesis is therefore focused on the fact that improvement in the survival rate in patients with curative surgery is due, amongst other factors, to the diagnosis of recurrence in early and asymptomatic stages that permit the curative resection of the recurrence. As a result, controversy exists with regard to the frequency at which these patients should be seen, which tests should be carried out, and if different follow-up strategies have an impact on the patient's progress [26].

Continuing on from the previous study [30], which allowed us to identify a cohort of 953 patients with colorectal cancer, we consider it to be of paramount interest to determine which variables from amongst those studied, as well as the different components of diagnosis delay, affect the prognosis of patients with cancer of the colon and rectum.

## Objectives

### Main objectives

▪ To determine if the symptoms-to-diagnosis interval and the treatment delay modify the survival rate of patients with cancer of the colon and rectum.

▪ To determine in patients with non-metastatic colorectal cancer treated with curative intention, if different follow-up strategies are associated with a higher survival rate.

### Secondary objectives

▪ To determine in patients with colorectal cancer:

a) The global survival rate.

b) Specific survival (mortality associated with the tumour)

c) Progression-free survival

d) Relapse-free survival

▪ From amongst the variables included in this study, to determine those which modify the prognosis in patients with colorectal cancer.

▪ To determine, in patients with non-metastatic colorectal cancer treated with curative intention, if different follow-up strategies are associated with a lower rate of local recurrence, the detection of asymptomatic recurrence, the detection of hepatic metastasis, and the rate of operable recurrences and metachronous tumours.

▪ To describe the variability in the follow-up of cancer of the colon and rectum according to the particular autonomous region and hospital, in terms of the types of tests used, the frequency at which the tests are used, and the specialist who carries out the follow-up.

## Methods/Design

Multi-centre prospective follow-up study in five regions of Spain (Galicia, Balearic Islands, Catalonia, Valencia and Aragón) during the period 2010-2012. It includes incident cases with anatomopathological confirmation of colorectal cancer according to the International Classification of Diseases (ICD) 9th revision (codes 153 and 154) which were included in the previous study [30] (n = 953). Prevalent or recurrent cases were excluded, together with cases of multiple cancer, cases that were only dealt with in private hospitals, cases detected through colorectal cancer screening, and cases diagnosed in another hospital but which were referred for treatment to the hospitals included in the study.

### Measurements

Information obtained from the previous study is available for each patient, which included variables related to the patient, the tumour (site, histological grade, TNM stage), clinical symptoms, delay intervals and characteristics of the health system in each of the regions studied [30]. Each of the patients was interviewed at the time of the diagnosis using a structured questionnaire. During the follow-up period, clinical records will be reviewed and surveillance of the outcomes of interest will be investigated.

The measurements that will be recorded on each patient at the time of diagnosis and during the follow-up can be found in Additional file 1: Table S1. Patients' variables include age, gender, civil status, educational level, family history of cancer, symptoms perception and the Charlson comorbidity index at the time of diagnosis. Tumour characteristics include: site (according to ICD-9^th^), tumour size, histological grade, TNM stage at diagnosis, location of metastases and infiltration of adjacent organs.

The symptoms-to-diagnosis interval is defined as the time elapsed from the date the patient perceived the first symptoms until the cytohistological confirmation of the diagnosis of cancer (date of biopsy or direct surgery). This delay has two components: patient delay and diagnosis delay (Table 1). Treatment delay is defined as the time elapsed between diagnosis and treatment. In this context we consider surgical treatment.

During the follow-up, the variables studied will be:

a) **Type of treatment**: in the surgical procedure we will investigate the type of resection, the need for a stent endoprosthesis and whether a colostomy is required or not. We will also take into account if planned or emergency surgery is performed, the curative or palliative intention of the surgery, the anastomosis technique, and if laparoscopic, open colorectal surgery, transanal resection and/or endoscopic resection is performed, as well as the need for reinterventions after the initial surgery.

The need and completion of chemotherapy and radiation therapy sessions will also be recorded.

b) **Hospital consultations related to the colorectal cancer and exploratory procedures**: in this section we account for the number of visits to the specialists (surgeons, digestive disease specialists and oncologists) and the diagnostic investigation procedures after the surgery. This information will be only collected on patients with non-metastatic colorectal cancer with curative intention. Among the diagnostic procedures, we consider imaging examinations (ecographies, positron emission tomographies, computed tomographies, magnetic resonances), endoscopy explorations (colonoscopies, rectoscopies) and carcionoembryonic antigen (CEA) determinations.

c) **Presence of incidents in the follow-up**: as outcome variables will be included local recurrence, development of metastases in the follow-up, appearance of a new tumour and mortality. The mortality records from each autonomous region will be used to determine the death of the patients and the cause, also determining whether the cause of death is associated with the colorectal cancer or not.

### Sample size

This study includes n = 953 patients. As we have two working hypotheses, one connected with diagnosis delay and another connected with the follow-up strategies, we will justify the sample size in order to deal with each of the hypotheses.

This sample size will make it possible to detect as significant, in a Cox regression model, a relative risk of 1.3 or more associated with greater delay, assuming an exposure to this possibility of 50% and a censored data percentage of 50% (Security: 95%; Statistical power: 80%). Assuming an exposure prevalence of 50% maximises the sample size necessary to detect a relative risk of this magnitude. In terms of the censoring value, we have estimated it at 50%, as according to published data [3] the estimated survival rate at five years for colorectal cancer in Spain is 61.2%. In this situation, the sample size required to estimate a relative risk of 1.3 or more (α = 0.05, β = 0.2) would be n = 912 patients.

Also, according to a revision of the Cochrane Library [31], proof was found that there are benefits for the global survival rate at five years for patients with colorectal cancer subjected to a more intensive follow-up (OR = 0.73; 95% IC = 0.59-0.91). This OR = 0.73 implies that patients without exhaustive follow-up would have a greater risk of death (1/0.73 = 1.4). In order to deal with the second objective, only patients with non-metastatic cancer receiving surgery with curative intention will be included. According to the data analysed so far for this cohort, approximately 17% of the patients would have a metastatic tumour, meaning that for the second objective 830 patients would be included. This sample size will make it possible to detect a risk of 1.4, with a censoring and exhibition percentage of 50%, and with the same security and power as in the previous case.

### Statistical analysis

Descriptive analyses will be performed for all variables. Continuous variables will be reported using means ± standard deviations (SD) or median (interquartile range). For dichotomous/categorical variables, absolute numbers and percentages will be computed, together with its 95% confidence intervals.

The comparison of means will be carried out using Student's t test, Mann-Whitney test, analysis of variance or the Kruskall-Wallis test as appropriate. The association of qualitative variables will be carried out using Chi-square statistics. The correlation among quantitative variables will be assessed using Spearman's correlation coefficient.

Actuarial survival analysis with Kaplan-Meier curves, log-rank test and Cox regression analysis will be performed. The assumption of hazards proportionality will be assessed using different procedures: a) a log-minus-log survival plot for each covariate, b) by analysis of scaled Schoenfeld residuals and c) by tests of interaction between categorized variables and time in the Cox model.

In case of violation of the hazard proportionality assumption for any of the covariates, an interaction term between that covariate and time will be included in the Cox regression model. Additionally, survival regression models using B-spline functions will be explored to model non-proportional hazards. These computations will be performed by using the functions available in the survival package in R (version 2.10.0).

Due to the fact that Kaplan-Meier method could overestimate the incidence of the events in the follow-up, a competitive mortality risk survival analysis will also be considered [32,33].

The analysis of the specific survival, progression-free survival and relapse-free survival rate will be carried out in a similar way to the analysis of the global survival rate. A similar analysis will be carried out to compare the prognosis of non-metastatic colorectal cancer subjected to surgery with curative intention according to the follow-up strategy.

The accuracy of different variables to predict events in the follow-up will be evaluated using the C-index [34]. Sensitivity, specificity and predictive values, with their 95% confidence intervals, will be determined for different cutoffs.

Two-sided tests will be used, and p-values < 0.05 will be considered as statistically significant. Statistical analyses will be performed using SPSS for Windows (version 17.0, SPSS Inc., Chicago, Illinois) and R (version 2.10.0).

Apart from performing an analysis of all of the patients, the patients with colon cancer and patients with rectal cancer will be analysed independently.

### Ethical and legal issues

The study will be carried out according to the good clinical practice guidelines of the Helsinki declaration. Informed consent was obtained from each patient to take part in the study and to review their clinical records. This project was approved by the ethics review board of each one of the sites participating in the study: Clinical Research Ethical Committee of Galicia (decision no. 2009/110), Clinical Research Ethical Committee of Illes Balears (decision no. 1167/09 PI), Clinical Research Ethical Committee of the Municipal Institute of Health Care (CEIC-IMAS, Barcelona) (decision no. 2009/3556/I), Clinical Research Ethical Committee of Aragón (decision no. 06/2009), and the Clinical Research Ethical Committee of the University Clinic Hospital of Valencia (date of approval June 5,2009).

## Discussion

Systematic reviews regarding the relationship between diagnosis delay and the degree of invasion and survival in colorectal cancer reveal the need to investigate this issue with larger samples of patients, and to analyse colon cancer and rectal cancer independently [21,22]. Some of the studies reviewed use specific stages or operable cases amongst their inclusion criteria, which introduce bias into the selection process and make it impossible to determine the relationship between the delay and the different stages in the diagnosis of colorectal cancer. Another problem that was observed was that in the majority of the studies, survival was calculated from the diagnosis or intervention date, instead of the date when the symptoms began, which could result in lead-time bias. These problems were taken into account in the protocol used in our study, where an independent analysis will be carried out depending on the location of the cancer in a cohort of nearly 1000 patients. This will include all of the incident cases diagnosed consecutively during the study period, which includes five different regions in the country. The study has excluded patients detected by screening and prevalent or recurrent cases, as in these cases the delay intervals are different. The study also excludes patients treated in private hospital, as in Spain the whole of the population is covered by the public health system.

The majority of studies use survival analysis strategies with the methodology of Kaplan and Meier. As this methodology may lead to overestimating the likelihood of the events of interest occurring, we propose a competitive risk analysis to study the prognosis of these patients [32,33].

Also, with regard to the follow-up strategies for patients with colorectal cancer, the ideal recommendations for this process have yet to be described [23]. These recommendations refer to the opinions of experts who indicate the performance of different examinations during the follow-up period [35,36]. Endoscopic techniques are the most frequently used, and imaging methods are the least used, with considerable variability amongst the different experts consulted [23]. Variability also exists depending on the geographic location of the experts consulted [37]. Other authors indicate that surveillance after potentially curative colon cancer surgery is not significantly affected by the geographic location of the surgeon who performs the surveillance testing, and only modestly affected by the population size of the metropolitan area in which he/she practices [31]. Carrying out multi-centre studies such as the one we propose, which studies variability in clinical practice in different regions of the same country, will provide more detailed information on its impact on the prognosis of these patients.

## Competing interests

The authors declare that they have no competing interests.

## Authors' contributions

SPF, SPD, ME, FM, MASC and AEM participated in the design and coordination of the study.

SPD, BLC and TSP are the biostatisticians of the study. AP provides informatic support for the project.

SPF, SPD, BLC, PGS, TSP, FAM, FM, MASC, AEM, MVA, AP, MRL, LGS, MRM, JMSN, IMA, LGL, MMR, CMN, OP, MMBW, ECI, MCA, MP, ACA, ART, ABP, CGA, MLB, SLM, MN, HM, CV, EF and ME reviewed the study protocol and made suggestions that improved the design. All of these individuals are involved in the management of the study.

SPF, SPD, BLC and TSP drafted the manuscript.

All of the authors read, revised and approved the final manuscript.

## Pre-publication history

The pre-publication history for this paper can be accessed here:

http://www.biomedcentral.com/1471-2407/10/528/prepub

## Supplementary Material

Additional file 1 **Table S1, Study measurements**. Table displaying the measurements that will be recorded on each patient at the time of diagnosis and during the follow-up. Click here for file
