# Supplementary material for: Diagnosis delay and follow-up strategies in colorectal cancer. Prognosis implications: a study protocol
Source: BMC Cancer. 2010 Oct 5;10:528. doi: 10.1186/1471-2407-10-528 (PMC2958943; doi:10.1186/1471-2407-10-528)
Supplement: Additional file 1 — Table S1, Study measurements. Table displaying the measurements that will be recorded on each patient at the time of diagnosis and during the follow-up. [file 1471-2407-10-528-S1.DOC]

**Supplementary Table 1. Study measurements at the time of diagnosis and during the follow-up.**

| **Patient variables** |
| --- |
| Age, gender, civil status, education, family history of cancer, symptom perception, Charlson comorbidity index |
| **Tumour** |
| Site, tumour size, histological grade, TNM at diagnosis, location of metastases, infiltration of adjacent organs |
| **Delay intervals** |
| - Symptoms-to-diagnosis interval: time elapsed from the date the patient perceived the first symptoms until the cytohistological confirmation of the diagnosis of cancer (date of biopsy or direct surgery). This delay has the following components:   - Patient-delay: Time elapsed from the date the patient perceived the first symptoms until the date of the first contact with a doctor as a result of the first symptom(s).   - Diagnosis delay: Time elapsed between the first contact with the health system until the diagnosis (date of the biopsy or direct surgery). - Treatment delay: Time elapsed between diagnosis and treatment. In this context we consider surgical treatment. Otherwise, chemotherapy or palliative care treatment as the first option. |
| **Treatment** |
| **Surgery** |
| Surgical procedures, planned or emergency surgery, laparoscopic or open colorectal surgery, type of surgical resection, curative or palliative surgery, type of anastomosis, visceral and metastases resection, surgical morbidity, reintervention, cause of the reintervention |
| **Chemotherapy** |
| Chemotherapy before and after surgery, number of cycles received |
| **Radiation therapy** |
| Radiation therapy before and after surgery, number of sessions received |
| **Follow-up** |
| **Hospital consultations** |
| **Imaging examinations (**ecographies, PETs^1^, CTs^2^, MRs^3^) |
| **Endoscopy explorations** |
| [**Carcinoembryonic antigen**](http://www.springerlink.com/index/H40T55K58441T033.pdf) **(CEA) determinations** |
| **Incidents in the follow-up** |
| **Local recurrence** |
| Diagnosis, location and treatment |
| **Development of metastases in the follow-up** |
| Diagnosis, location and treatment |
| **Appearance of a new tumour** |
| Diagnosis, location and treatment |
| **Mortality** |
| Cause of death |

^1^PET: Positron Emission Tomography; ^2^CT: Computed tomography; ^3^MR: magnetic resonance
